# Psychometric Assessment of the Romanian Version of the Index of Dental Anxiety and Fear (IDAF-4C^+^)

**DOI:** 10.3390/healthcare11152129

**Published:** 2023-07-26

**Authors:** Alexandra Elena Done, Elena Preoteasa, Cristina Teodora Preoteasa

**Affiliations:** 1 Department of Scientific Research Methods-Ergonomics, Faculty of Dentistry, “Carol Davila” University of Medicine and Pharmacy, 020021 Bucharest, Romania; cristina.preoteasa@umfcd.ro; 2 Department of Prosthodontics, Faculty of Dentistry, “Carol Davila” University of Medicine and Pharmacy, 020021 Bucharest, Romania

**Keywords:** IDAF-4C^+^, DAS, dental anxiety

## Abstract

Background and Objectives: The goal of this study was to assess the validity of the Romanian version of the Index of Dental Anxiety and Fear (IDAF-4C^+)^ questionnaire. Materials and Methods: This study was conducted on a convenience sample of past patients and their acquaintances through an online questionnaire administered on the Google Forms platform between May 2021 and September 2022. The sections of the survey were demographic characteristics, the dental anxiety scale questionnaire, the IDAF-4C^+^ questionnaire, a single question about dental fear, and previous dental treatments. Results: In total, 239 participants were included in the study, and the mean age was 37. The IDAF-4C questionnaire had good internal validity (Cronbach alpha was 0.945). The IDAF-4C had good convergent validity, and it was positively correlated with the dental anxiety scale (r = 0.825, *p* < 0.001) and the question about the fear of going to the dentist (r = 0.738, *p* < 0.001). The questionnaire had good reliability, and the intraclass correlation was 0.985. Lower levels of dental anxiety were associated with scaling, orthodontic treatment, and dental implants. A confirmatory factor analysis was conducted after the removal of the first question from the phobia module, and residual covariance was added between items four and nine of the stimulus module, showing a good fit for the retained questions of the IDAF-4C^+^, grouped by module. Conclusions: The Romanian version of the IDAF-4C^+^ showed acceptable psychometric properties.

## 1. Introduction

Dental anxiety is a shared problem among patients; according to a systematic review, dental anxiety affects 15.3% of adults worldwide [1]. Dental anxiety or dental fear are emotions that patients can experience related to dental treatment. In general, anxiety and fear are responses to a threat. Those terms are frequently used interchangeably. The difference between them is that anxiety is a response to a potential danger that could happen in the future, while fear is a response to a specific stimulus in the present. A phobia is a high-intensity fear, without a reason, of a situation or an object that leads to avoidance or marked distress [2].

In Romania, there are few studies that evaluate the level of dental anxiety. According to them, 4.3% of Romanian participants from Iasi were highly anxious, and 2.9% were extremely anxious [3]. In another study conducted in Cluj-Napoca on deaf people, 59.7% of the participants had moderate or very high levels of dental anxiety, and 5.3% had a dental phobia [4]. For children 11 to 18 years old, 42.61% had moderate dental fear, and 10.84% were susceptible to a dental phobia; the questionnaire used was the dental fear survey [5].

Dental anxiety has negatively influenced oral health, as patients who are afraid of going to the dentist usually postpone dental appointments; therefore, their oral condition becomes worse, and they present to the dentist mostly with an emergency. This behavior leads to a vicious cycle because they can have pain before the procedure and need complex treatments. Due to the association of pain with dental treatment, it is likely that patients will postpone the needed follow-up appointment until they have another emergency [6,7].

Among the methods used in the literature, questionnaires are frequently used to recognize dental anxiety. The dental anxiety scale is a short form with four questions, and it is a rapid and uncomplicated way to recognize patients’ levels of anxiety [8]. Armfield [9] developed another questionnaire, the Index of Dental Anxiety and Fear (IDAF-4C^+^), which assesses the level of dental anxiety, the stimuli that determine dental anxiety, and the presence of a dental phobia. In terms of the level of anxiety, Armfield had an approach that is more complex than the other methods used before. In the section which measures the level of dental anxiety (the core module, placed first, named the IDAF-4C), the behavior, emotional, cognitive, and physiological components of dental anxiety are recorded, not only the emotional aspect frequently addressed in other questionnaires. This questionnaire, the IDAF-4C^+^, was not validated in the Romanian language previously.

The aim of this study was to validate the Romanian version of the IDAF-4C^+^ and to evaluate its validity and reliability. We also aimed to see how previous dental treatment experience related to dental fear and anxiety.

## 2. Materials and Methods

This study was approved by the Research Ethical Committee of the University of Medicine and Pharmacy “Carol Davila” from Bucharest.

### 2.1. Study Design and Sample

This cross-sectional study was conducted on a convenience sample of people with a past history of dental treatment from Romania. Past patients from the dental clinic and acquaintances were invited to participate in this research and also to distribute the questionnaire to 5 of their acquaintances. This invitation was sent via WhatsApp or e-mail, and data were not collected in the dental office. The inclusion criteria were age 18 or over and willingness to respond to the survey. People that had never been to the dentist were excluded. For sample size, a respondent-to-item ratio of 10:1 was chosen [10]; therefore, sample size of 230 participants was targeted. Study inclusion was performed from May 2021 to September 2022 until the desired sample size was reached. An informed consent form was placed at the beginning of the questionnaire sent online. Data in it included information on not being recompensed for participation in this study.

### 2.2. Variables and Data Collection

Data were collected through an online survey on Google Forms platform.

The survey was divided into four sections:

- demographic characteristics (i.e., age, gender, and level of education);
- dental anxiety level;
- previous dental treatments (scaling, orthodontics, removable dentures, fixed prosthetics, endodontics, dental extractions, or dental implants);
- when their last visit to the dentist was (6 months or more).

The level of dental anxiety was recorded with the dental anxiety scale questionnaire (DAS), the Index of Dental Anxiety and Fear (IDAF-4C^+^), and a single-question assessment of dental anxiety (“In general, are you afraid of going to the dentist? Response options: not at all, slightly, moderately, very, very much”).

The dental anxiety scale (DAS) questionnaire is a short questionnaire that was developed by Corah [11] in 1969, and it is a widely used questionnaire for dental anxiety [1]. There are four questions; the first two questions of the DAS questionnaire record the anxiety one day before the treatment or just before the beginning of the treatment, and the next two questions record the anxiety during two common dental treatments. Each question has five answers ranging from 1 to 5, and the score of the DAS is the sum of the answers. According to DAS, the level of dental anxiety is considered low for values under 9, moderate for values between 9 and 12, high for values 13 and 14, and extremely high for values over 15 [8].

IDAF-4C^+^ has three modules: the core, stimulus, and phobia. The core module has eight questions which are designed to capture the level of fear and anxiety by measuring the emotional, behavioral, physiological, and cognitive aspects of dental anxiety. The level of anxiety measured by IDAF-4C is determined as the average of the answers to these eight questions. Each question is rated from 1 to 5 on a Likert scale. According to IDAF-4C, the level of anxiety is low for values from 1 to 1.5, moderate from 1.5 to 3.5, and severe for values higher than 3.5 [12].

The stimulus module includes 10 questions that record the main stimuli causing dental anxiety on a five-point Likert scale (i.e., pain, lack of control, the cost of the treatment, injections or needles, numbness caused by the anesthetic, feeling sick, feeling embarrassed, unkind dentist, gagging, and not knowing what the dentist is going to do) [9].

The phobia module has five dichotomic questions (yes/no). The first three questions investigate if going to the dentist is avoided in an active way, for how long this fear has been present (e.g., more than 6 months), and if it has a negative impact on daily life. The next two questions investigate the fear of having a panic attack and the fear of being watched in social situations as potential alternatives to dental phobia in explaining the fear of going to the dentist [9].

Translation of IDAF-4C^+^. Translation of the IDAF-4C^+^ was carried out using the forward–backward method. Firstly, the forward translation of the source version from English into Romanian was conducted independently by two translators. Discrepancies were resolved afterward by translators, and the first Romanian draft was obtained. Secondly, blind back translation was performed, from Romanian into English, by two other translators. Thirdly, the final Romanian version was generated by the team after a review of all previous versions of forward and backward translations. All translators are native Romanian speakers, proficient English speakers, and have a dental degree.

### 2.3. Statistical Analysis

The internal consistency of the IDAF-4C^+^ anxiety and fear module was evaluated using Cronbach alpha, inter-item correlation analysis, Corrected Item-Total Correlation, and Cronbach alpha if the item was deleted.

For the test–retest reliability, intraclass correlation was performed. For assessment of test–retest reliability, the questionnaire was sent again to the participants, with the interval between the two time points being 4 weeks.

Cohen’s Kappa coefficient was calculated to determine if there was an agreement between the IDAF-4C and the DAS. The cut-off points for IDAF-4C and DAS were 3 and 13, respectively [12].

The differences among groups were evaluated using the Chi-square test for dichotomous variables and the Mann–Whitney test for quantitative variables, as data were not normally distributed.

For confirmatory factor analysis, the JASP and R program were used. DWLS estimator was used because of ordinal and nominal variables [13]. The reported fit indices were: Chi-square test, Root means square error of approximation (RMSEA), Comparative Fit Index (CFI), and Tucker–Lewis Index (TLI). Confirmatory factor analysis was first conducted separately, with the one-factor model being performed for each of the three modules. Afterward, a three-factor model was performed for all IDAF-4C^+^ modules (core, stimulus, and phobia modules). In the next step, an inspection of modification indices was performed, followed by removal of the first question from the phobia module. Otherwise, the model would not be admissible. This question was found to fit better with the IDAF-4C (the core module). A residual covariance was added between item 4 (feeling sick, queasy, or disgusted) and item 9 (gagging or choking) from the stimulus module after the inspection of modification indices.

SPSS Statistics was used for data analysis except for confirmatory factor analysis. The level of statistical significance was *p* < 0.05.

## 3. Results

The study sample included 239 participants. The link was sent to 75 past patients, of which 68 responded. In addition to the previous respondents, 171 of their acquaintances were included. Most of them were females (n = 158; 66.11%); the mean age was 37 years (min 18, max 88, standard deviation 15.12).

Cronbach alpha for the IDAF-4C anxiety and fear module was 0.945, which indicates excellent internal consistency. Removal of any items would not improve the value of Cronbach alpha. The items were strongly associated with one another, and the correlation coefficient had values between (0.61 and 0.82) (Table 1).

IDAF-4C anxiety and the fear module were highly correlated with DAS and the single question for dental fear assessment (Table 2).

There was a moderate agreement between IDAF-4C and DAS, Cohen’s kappa coefficient (κ) = 0.617, *p* < 0.001. Almost all participants identified as having dental anxiety by DAS were also identified by IDAF-4C, but of those identified as having dental anxiety by IDAF-4C, only nearly half were also identified as having dental anxiety by DAS (Table 3).

Test–retest reliability analysis indicated that the intraclass correlation coefficients were 0.985 for IDAF-4C, 0.89 for DAS, and between 0.519 and 0.906 for the stimulus module. (Table 4).

Analyzing the IDAF-4C anxiety and fear module and the stimulus module in relation to dental treatments, it was found that participants who had scaling, implant insertion, and orthodontic treatment had a lower level of anxiety compared with those who had not. Participants who had treatment with removable dentures had a higher level of anxiety than those who had not (Table 5).

Pain during treatment, costs of dental treatment, and using needles and injections were considered the stimuli that generate the highest anxiety for patients in relation to dental visits. The cost of treatment had a weak association with the IDAF-4C^+^ score (Table 5). There were some differences between males and females. Overall, except for the cost of the treatment, women had higher values for dental stimuli than men, but the difference was statistically significant only for gagging, having an unsympathetic dentist, and feeling sick (Table 6). Participants who visit the dentist for regular check-ups had statistically significantly lower values reported for all dental stimuli than those who do not go for regular check-ups (Table 6).

All the participants who responded “Yes” to the phobia module had a higher level of anxiety than those who responded “No”. The mean values of the IDAF-4C scores were between (2.2 and 2.79). According to the phobia module in relation to dental treatments, participants who had dental scaling had a lower chance of responding “yes” to all phobia items; the difference was statistically significant. Participants who had orthodontic treatment had a lower chance of responding “yes” to “going to the dentist is actively avoided”, “fear of going to the dentist significantly affects my life”, and “I am afraid of going to the dentist because I am concerned I may have a panic attack”; the differences were statistically significant. Participants who had dental implants had lower a chance of responding affirmatively to “going to the dentist is actively avoided”; the difference was statistically significant. Participants who had removable denture treatment had a higher chance of responding “yes” to “fear of going to the dentist significantly affects my life”. (Table 7).

According to the confirmatory factor analysis, each module, i.e., the IDAF-4C core module, the phobia module, and the stimulus module, had a good fit. The Comparative Fit Index and Tucker–Lewis Index were higher than 0.994 and the RMSEA < 0.05 for all modules (Table 8).

**Figure 1:**
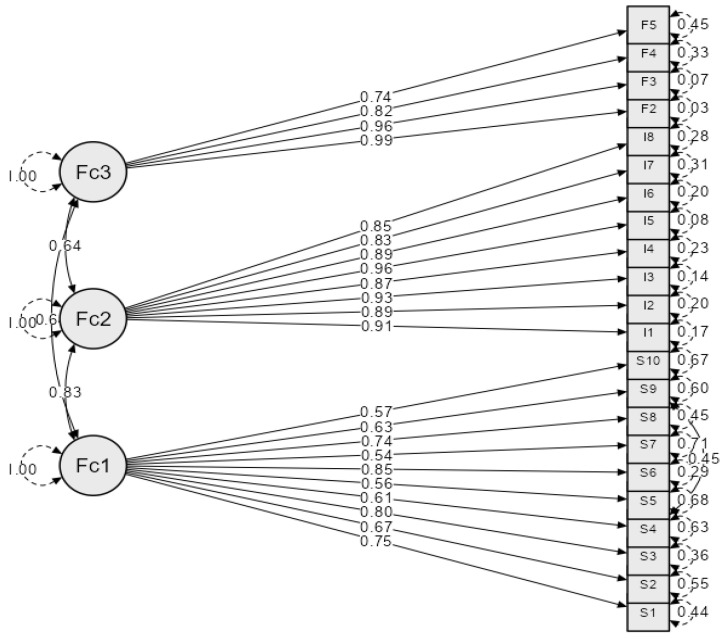
A path diagram of confirmatory factor analysis of IDAF-4C^+^ modified by the removal of the first question from the phobia (F2–F5 represent phobia module items, I1–I8 core module items, and S1–S10 stimulus module items).

Results of the confirmatory factor analysis for IDAF-4C^+^, modified in regard to the removal of the first question from the phobia module due to the reasons previously mentioned, with questions grouped in three factors, are presented in Table 8 and Figure 1.

## 4. Discussion

IDAF-4C had good internal validity (Cronbach alpha was 0.945), and good convergent validity was as shown by the positive correlation with the dental anxiety scale (r = 0.825, *p* < 0.001) and the question regarding the fear of going to the dentist (r = 0.738, *p* < 0.001). There was a moderate agreement between the IDAF-4C and the DAS questionnaires (κ = 0.617). Furthermore, IDAF-4C had good reliability, and the intraclass correlation was 0.985.

Similarly to Armfield [9], participants who responded “yes” to phobia module items had a higher mean IDAF-4C than those who responded “no”. However, the mean IDAF-4C of the participants who responded “yes” in this article was lower than 3 (i.e., between 2.2 and 2.79), which, except for social anxiety, is in contrast to Armfield’s findings [9] (their IDAF-4C mean was above 3). However, the phobia module is a screening and not a diagnostic tool [9].

From the myriad dental treatments suspected to change the level of dental anxiety, we selected the following seven: scaling, orthodontics, removable denture, fixed prosthetics, endodontics, dental extractions, and dental implants. These treatments were selected based on our ex ante expectation regarding their frequency in our study sample and their notoriety among our participants (i.e., suggesting our participants will be familiar with them when answering the questionnaire).

According to this research, previous experiences with dental treatments seem to be linked to different levels of dental anxiety. We found that lower levels of dental anxiety are associated with scaling, orthodontic treatment, and dental implants. Higher levels of dental anxiety are associated with removable dentures, while endodontic treatment, tooth extraction, and fixed dental restoration are not related to the level of anxiety. When interpreting these results, the cross-sectional nature of our study suggests an association relationship, not a causal one. Some dental treatments may contribute to reducing the level of dental anxiety, but the level of dental anxiety may also affect the patient’s choice of dental treatment. This is consistent with findings from the control groups of randomized controlled trials showing improvement in dental anxiety levels after dental intervention [14,15]. This is explicable, considering that by accessing dental treatment subjects understand better what is happening during the procedures and become familiar with it. Previous research shows a decrease in anxiety levels during orthodontic treatment [16]. The decrease in anxiety levels can be explained by increased familiarity with orthodontic treatments (due to regular visits to the dental office), low levels of pain and discomfort during the treatment, and increased understanding of treatment risks and complications [17]. Also, consistent with the literature, anxiety decreased after scaling, suggesting that it is an anxiety-protective factor [18,19,20]. Scaling may contribute to reducing dental anxiety, but it is also possible that patients with lower levels of dental anxiety tend to access this type of dental procedure more often. From our results, the level of dental anxiety was not associated with fixed dental restoration, probably because an association was difficult to be found due to their increased variability, but on the contrary, removable prosthodontic treatment was associated with a higher anxiety level. Participants with dentures had a higher level of anxiety. The IDAF-4C score was 2.47 for participants with dentures and 1.71 for those without; the difference was statistically significant (*p* = 0.004). This finding is probably best explained by the fact that dental anxiety is a barrier to seeking dental care with a negative impact on oral health [21], and also that people with a high level of dental anxiety prefer to have their teeth extracted than restored [6,22]. Therefore, it is suggested that people with a high level of dental anxiety are at risk to become wearers of removable prostheses. This alternative is not always the most indicated when compared with other variants, in regard to several aspects such as oral health-related quality of life or masticatory performance [23,24,25]. In this study, participants who had removable denture treatment had a higher chance of responding affirmatively to “fear of going to the dentist significantly affects my life in some way (dental pain, avoiding eating some foods, embarrassed or self-conscious about appearance of teeth or mouth, etc.)”. To improve the quality of life of participants who need removable dentures, dentists can consider some aspects to improve the treatment, for example, the improvement of stability.

Our results, in conjunction with prior evidence, suggest that prospective studies are recommended to clarify to what extent some dental intervention with good acceptability, such as scaling, contributes to lowering dental anxiety levels. Also, it has to be established which are the main dental treatments avoided by patients due to their high dental anxiety levels. This knowledge is important for better control of oral health problems.

The dental anxiety scale, DAS, and single-question assessment were highly correlated with the emotional component of the IDAF-4C (r = 0.827, *p* < 0.001, and r = 0.727, *p* < 0.001), similar to previous research [9,12,26,27]. Armfield [9], had similar results; the emotional component was highly correlated with the DAS (r = 0.83) and with a single item of dental fear (r = 0.59). He sustained that this correlation was expected because of the emotional aspect of the DAS and a single item of dental fear.

Similar to other studies, the lowest correlation between the dental anxiety scale and single-question assessment was with the cognitive aspect of the IDAF-4C [9,28]. However, for the single-question assessment, a higher correlation was found in this research (r = 0.623, *p* < 0.001) than in Armfield’s [9] study (r = 0.37, *p* < 0.001).

There were some discrepancies between the level of anxiety recorded by the IDAF-4C and the DAS questionnaire. In this study, more participants reported anxiety with the IDAF-4C (n = 31) than with the DAS (n = 18). Only 16 participants were identified with anxiety by both questionnaires. Ibrahim [29] reported different results; fewer participants were linked to having dental anxiety by the IDAF-4C (n = 33) than with the DAS (n = 47), and 23 had anxiety according to both questionnaires. The agreement between the two indices in this study (κ = 0.617, *p* < 0.001) was higher than that in Ibrahim (κ = 0.498) [29]. Discrepancies between the level of anxiety recorded by the Modified dental anxiety scale (MDAS) and the IDAF-4C were found also in the validation study for Finnish and Italian participants, maybe because of the cut of point(s) that were arbitrarily established [26,27].

The IDAF-4C had a good internal validity. Cronbach alpha was 0.945, and the value was similar to that in previous research, where this coefficient had a value between 0.80 and 0.96 [30,31].

By the results of the confirmatory factor analysis, conducted by us, it is suggested that the IDAF-4C+ has an acceptable structure; however, some modifications need to be assessed if they are appropriate. Firstly, it should be revised if question one from the phobia module requires rephrasing or if removing it would be appropriate. Secondly, from our results, item 4 (“Feeling sick, queasy or disgusted”) and item 9 (“Gagging or choking”) from the stimulus module should be revised, as a residual covariance between them was found. These findings may be related to the perception of the persons who filled the questionnaire that these items are linked or overlap to some extent, e.g., gagging may be accompanied by nausea, feeling sick, or queasy. Also, these two items refer to sensations well-known to be possible to experience during dental treatment, being also associated with dental anxiety and phobia [32,33]. To our knowledge, confirmatory factor analysis was previously conducted only for the IDAF-4C (core module) [31,34], but not for the IDAF-4C^+^. Therefore, it would be recommended to conduct it for IDAF-4C^+^, preferably for the original English version of this instrument.

In the literature, it was reported that women had a higher level of anxiety than men [12,35]. In our study, we observed that females are only anxious related to specific stimuli (i.e., feeling sick, gagging, and having an unsympathetic dentist).

Dental stimuli were positively associated with the IDAF-4C core module score; the strongest association was with the item “not knowing what the doctor is going to do”, and the weakest association was with the item “cost of dental treatments”. The results are similar to those obtained in a research conducted on a sample of Malaysian participants [31].

Participants who regularly go to the dentist reported a lower level of pain associated with dental anxiety than those who do not. Similarly, Furgala et al. [36] consider that pain is an important stimulus for dental anxiety and reducing it will improve attendance to dental treatment.

Regular visits to the dentist were associated with a lower concern about stimulus from the dental fear module; these results were similar to those found in the research of Carrillo-Diaz et al. [37], which conducted the validation of the Spanish version of the IDAF-4C. The response on item “Not knowing what the dentist is going to do” was highly correlated with the level of anxiety measured by the IDAF-4C. Therefore, improvement of the given information to participants should be considered for reducing dental anxiety. Correct information, adapted to individual levels of comprehension, should be given to patients [38].

Overall, the IDAF-4C^+^ can be seen as a tool that has good psychometric properties, but also its shortcomings should be acknowledged when used. Firstly, it should be noted that its theoretical base is relatively well-defined and it is argued that what is being assessed is clear. This was perceived as being deficient in some of the previous tools used for the same purpose [9,29]. Even so, it should be noted that it targets concepts that partially overlap and are also relatively hard to differentiate by the means of a questionnaire, i.e., fear, anxiety, and phobia. In regard to the first two terms, fear and anxiety, it can be argued that even if these concepts are basically different, they are often used interchangeably, which may be one reason for presenting them as a single item. One main difference between fear and anxiety is related to the threat characteristics, with the first being a present-oriented response to a perceived threat, while the second is a future-oriented response to a diffuse threat [2]. The previous may also be a reason why both terms were used, as well as the questionnaire being possibly given to patients that will receive dental services in the near future, or to people that do not have a dentist appointment but are conscious that they will probably go at some point in time. In regard to the third term, phobia refers to excessive fear, which is associated with strenuous avoidance or endurance of dental treatment [2] and poses a greater impact on attending dental services or challenges in conducting dental treatment [39]. Even if the overlapping of these concepts is an issue, the step forward in differentiating patients with a dental phobia from those with dental anxiety should be acknowledged. Also, the structure of the questionnaire should be noted, with modules that target different aspects of dental anxiety. The core module of the IDAF-4C comprises clearly defined directions for the assessment of dental anxiety and assesses the overall level of fear [29], being meant to differentiate between persons with low, moderate, or high dental fear, being designed as a scale. The core module can be used per se, or together with the other two modules. The phobia module, placed second in the structure of the questionnaire, addresses some shortcomings of some previous tools, which rarely took into consideration, for example, the avoidance of accessing dental treatment [30]. The phobia module has a solid theoretical and psychological base, as its conception started from diagnostic criteria for a specific fourth edition of the Diagnostic and Statistical Manual of Mental Disorders [9]. It was not designed as a scale; therefore, it has some limitations of usage in research, this being an aspect that can be improved. It was probably designed to be used additionally to the first one, depending on the results obtained. A score higher than three in the core module was considered diagnostic criteria for phobia, [9] but it needs further research for confirmation. In our sample, the score mean of the IDAF-4C core module was lower than three for each item of the phobia module.

The third module had a specific focus on dental feared stimuli, and it was not designed as a scale index as well. Overall, it assesses a complex condition by means of a short-form questionnaire It has advantages compared with other tools, which may make it more appropriate to be used in some research and also in clinical practice. In our opinion, the IDAF-4C^+^ presents an improvement compared with other tools made for similar purposes and can be also a base for developing this difficult-to-assess and relatively frequent encountered aspect.

The main study limitations were: the usage of convenience sampling, potentially introducing selection bias that limits the generalizability of the findings; the cross-sectional nature of the study; and a prospective design indicated for some of the aspects investigated as the relation between dental anxiety and different treatments. Another study limitation was the decreased number of dental treatments considered. More dental treatments should have been considered for a better understanding of anxiety in dental practice. We chose only seven treatments that are associated with different levels of dental anxiety. We tried to develop a short list of dental treatments that are well-known by the general population and easy to recognize in the questionnaire.

## 5. Conclusions

The Romanian version of the IDAF-4C^+^ showed acceptable psychometric properties, but ways of improvement of the phobia and stimulus modules could be considered.

## Figures and Tables

**Table 1:** Inter-item correlation matrix of IDAF-4C anxiety and fear module.

Item	Mean	Inter-Item Correlation Matrix								Corrected Item-Total Correlation	Cronbach Alpha If Item Deleted
		I1	I2	I3	I4	I5	I6	I7	I8		
I1	2.03	-	0.74	0.73	0.64	0.82	0.74	0.62	0.68	0.83	0.935
I2	1.78		-	0.76	0.64	0.72	0.62	0.69	0.63	0.80	0.938
I3	1.64			-	0.74	0.78	0.74	0.65	0.62	0.84	0.936
I4	1.44				-	0.71	0.63	0.61	0.63	0.76	0.941
I5	1.74					-	0.84	0.69	0.74	0.89	0.931
I6	1.73						-	0.62	0.67	0.81	0.937
I7	1.95							-	0.64	0.75	0.943
I8	1.67								-	0.76	0.940

**Table 2:** Spearman’s Rho between the IDAF-4C, IDAF-4C components (emotional, behavioral, cognitive, physiologic), dental anxiety scale (DAS), and single-question assessment of dental anxiety.

Measure	2	3	4	5	6	7
1. emotional	0.750 **	0.748 **	0.823 **	0.926 **	0.827 **	0.727 **
2. behavior		0.727 **	0.689 **	0.882 **	0.685 **	0.638 **
3. cognitive			0.737 **	0.841 **	0.681 **	0.623 **
4. physiologic				0.878 **	0.753 **	0.676 **
5. IDAF-4C					0.825 **	0.738 **
6. DAS						0.775 **
7. single-question assessment						

** *p* < 0.001.

**Table 3:** Agreement between IDAF-4C and DAS.

		DAS		
		<13	≥13	Total
IDAF-4C	<3	206 (83.2%)	2 (0.8%)	208 (87%)
	≥3	15 (6.3%)	16 (6.7%)	31 (13%)
	Total	221 (92.5%)	18 (7.5%)	239 (100%)

**Table 4:** Intra-class correlation for IDAF-4C, DAS, and stimulus module (I1–I10).

	Intra-Class Correlation	95% Confidence Interval		*p*
IDAF-4C	0.985	0.956	0.995	<0.001
DAS	0.894	0.683	0.964	<0.001
I1	0.814	0.445	0.937	0.002
I2	0.813	0.442	0.937	0.002
I3	0.581	−0.248	0.859	0.058
I4	0.776	0.332	0.925	0.004
I5	0.735	0.211	0.911	0.009
I6	0.855	0.567	0.951	<0.001
I7	0.906	0.719	0.968	<0.001
I8	0.86	0.584	0.953	<0.001
I9	0.519	−0.432	0.839	0.092
I10	0.819	0.462	0.939	0.001

**Table 5:** IDAF-4C^+^ core score and stimulus module results in accordance with the participant’s past dental treatment experience.

IDAF-4C^+^ Component	Scaling	Endodontics	Tooth Extraction	Implants	Orthodontics	Removable Dentures	Fixed Dentures
	No/Yes (Mean); (*p*)	No/Yes (Mean); (*p*)	No/Yes (Mean); (*p*)	No/Yes (Mean); (*p*)	No/Yes (Mean); (*p*)	No/Yes (Mean); (*p*)	No/Yes (Mean); (*p*)
IDAF-4C core score	2.07/1.62 (<0.001)	1.745/1.747 (0.883)	1.82/1.69 (0.403)	1.78/1.45 (0.022)	1.81/1.47 (0.003)	1.71/2.47 (0.004)	1.71/1.8 (0.178)
IDAF-4C^+^—stimulus module:							
I1 (pain)	3.01/2.85 (0.322)	2.83/2.97 (0.390)	2.78/2.99 (0.149)	2.93/2.65 (0.260)	2.93/2.78 (0.507)	2.89/3.08 (0.583)	2.85/3 (0.328)
I2 (embarrassed)	2.12/1.55 (0.001)	1.66/1.75 (0.327)	1.80/1.64 (0.484)	1.74/1.42 (0.032)	1.76/1.51 (0.243)	1.66/2.46 (0.009)	1.67/1.80 (0.416)
I3 (no control)	2.32/1.965 (0.01)	2.06/2.08 (0.995)	2.17/1.99 (0.33)	2.09/1.92 (0.665)	2.13/1.82 (0.077)	2.04/2.54 (0.083)	2.03/2.13 (0.379)
I4 (feeling sick)	1.64/1.31 (<0.001)	1.38/1.43 (0.733)	1.44/1.38 (0.29)	1.42/1.27 (0.409)	1.43/1.31 (0.299)	1.39/1.62 (0.041)	1.39/1.43 (0.595)
I5 (numbness)	2.16/1.72 (0.001)	1.92/1.77 (0.202)	2.05/1.69 (0.018)	1.86/1.73 (0.778)	1.93/1.53 (0.013)	1.84/1.92 (0.425)	1.84/1.86 (0.774)
I6 (not knowing)	2.29/1.93 (0.01)	1.97/2.1 (0.535)	2.19/1.91 (0.055)	2.05/1.89 (0.617)	2.11/1.74 (0.03)	2.01/2.46 (0.085)	1.97/2.16 (0.104)
I7 (cost)	3.12/2.64 (0.009)	2.78/2.77 (0.956)	2.79/2.76 (0.939)	2.80/2.58 (0.468)	2.82/2.59 (0.317)	2.73/3.54 (0.031)	2.74/2.83 (0.621)
I8 (injection)	2.84/2.33 (0.003)	2.56/2.38 (0.23)	2.54/2.42 (0.557)	2.52/2.08 (0.14)	2.57/2.10 (0.03)	2.43/3.23 (0.037)	2.42/2.58 (0.335)
I9 (gagging)	1.72/1.59 (0.138)	1.64/1.6 (0.54)	1.66/1.6 (0.171)	1.67/1.27 (0.068)	1.66/1.49 (0.369)	1.62/1.77 (0.149)	1.60/1.66 (0.458)
I10 (unsympathetic dentist)	2.33/1.88 (0.014)	1.96/2.06 (0.57)	1.95/2.05 (0.656)	2.08/1.46 (0.025)	2.05/1.84 (0.198)	2/2.15 (0.878)	2.03/1.98 (0.665)

**Table 6:** Stimulus module—mean scores, correlation with IDAF-4C core score, and difference according to sex and attendance of regular check-ups.

	Total (Mean Score)	Association with IDAF-4C (*p*)	Male (Mean Score)	Female (Mean Score)	*p*	Check-Ups No (Mean Score)	Check-Ups Yes (Mean Score)	*p*
I1 (pain)	2.9	0.556 (*p* < 0.001)	2.77	2.97	0.19	3.109	2.565	<0.001
I2 (embarrassed)	1.71	0.429 (*p* < 0.001)	1.57	1.78	0.11	1.912	1.380	<0.001
I3 (no control)	2.07	0.568 (*p* < 0.001)	1.88	2.16	0.05	2.197	1.859	0.009
I4 (feeling sick)	1.41	0.344 (*p* < 0.001)	1.27	1.47	0.03	1.537	1.196	<0.001
I5 (numbness)	1.85	0.336 (*p* < 0.001)	1.73	1.91	0.34	2.014	1.576	<0.001
I6 (not knowing)	2.03	0.636 (*p* < 0.001)	1.99	2.06	0.55	2.245	1.696	<0.001
I7 (cost)	2.77	0.328 (*p* < 0.001)	2.79	2.77	0.87	3.034	2.359	<0.001
I8 (injection)	2.47	0.558 (*p* < 0.001)	2.3	2.56	0.09	2.680	2.141	<0.001
I9 (gagging)	1.62	0.411 (*p* < 0.001)	1.44	1.72	0.02	1.782	1.370	<0.001
I10 (unsympathetic dentist)	2.01	0.421 (*p* < 0.001)	1.74	2.15	0.02	2.109	1.848	0.037

**Table 7:** Comparisons among IDAF-4C^+^ phobia module (I1–I5) scores with different previous dental treatments.

			IDAF-4C Mean	Scaling	Endodontics	Tooth Extraction	Implants	Orthodontics	Removable Dentures	Fixed Dentures
				Yes/No	Yes/No	Yes/No	Yes/No	Yes/No	Yes/No	Yes/No
				172/67	120/119	137/102	26/213	49/190	13/226	83/156
I1	Yes	66 (27.6%)	2.79	39/27	30/36	34/32	2/64	8/58	6/60	25/41
	No	173 (72.4%)	1.35	133/40	90/83	103/70	24/149	41/132	7/166	58/115
	*p*		<0.001	0.006	0.223	0.165	0.01	0.032	0.114	0.314
I2	Yes	51 (21.3%)	2.66	24/27	25/26	27/24	3/48	6/45	5/46	19/32
	No	188 (78.7%)	1.5	148/40	95/93	110/78	23/165	43/145	8/180	64/124
	*p*		<0.001	<0.001	0.487	0.289	0.148	0.56	0.118	0.393
I3	Yes	42 (17.6%)	2.6	19/23	22/20	22/20	3/39	4/38	6/36	17/25
	No	197 (82.4%)	1.56	153/44	98/99	115/82	23/174	45/152	7/190	66/131
	*p*		<0.001	<0.001	0.444	0.293	0.291	0.036	0.014	0.245
I4	Yes	22 (9.2%)	2.28	7/15	10/12	9/13	2/20	1/21	2/20	8/14
	No	217 (90.8%)	1.69	165/52	110/107	128/89	24/193	48/169	11/206	75/142
	*p*		0.002	<0.001	0.404	0.08	0.562	0.036	0.341	0.518
I5	Yes	31 (13%)	2.22	15/16	16/15	13/18	2/29	3/28	3/28	10/21
	No	208 (87%)	1.68	157/51	104/104	124/84	24/184	46/162	10/198	73/135
	*p*		<0.001	0.003	0.51	0.049	0.312	0.08	0.229	0.463

**Table 8:** Fit indices for the core module (IDAF-4C), phobia module, stimulus module, and modified IDAF-4C^+^.

Fit Indices	Core Module (IDAF-4C)	Phobia Module	Stimulus Module	Modified IDAF-4C^+^
Chi-square test χ^2^(df); *p*-value	28.111 (20); *p* = 0.107	5.783 (5); *p* = 0.328	52.857 (34); *p* = 0.021	255.353 (205); *p* = 0.01
Comparative Fit Index (CFI)	1	0.999	0.995	0.998
Tucker–Lewis Index (TLI)	0.999	0.999	0.994	0.998
Root means square error of approximation (RMSEA)	0.041	0.026	0.048	0.032
RMSEA 90% CI lower bound	0	0	0.019	0.017
RMSEA 90% CI upper bound	0.074	0.097	0.073	0.044
RMSEA *p*-value	0.628	0.626	0.517	0.995

## Data Availability

The data presented in this study are available on request from the corresponding authors.
